# Serum concentration of ferroportin in women of reproductive age

**DOI:** 10.11613/BM.2024.030701

**Published:** 2024-08-05

**Authors:** Ana Ćuk, Lada Rumora, Ivanka Mikulić, Nikolina Penava, Ivona Cvetković, Ante Pušić, Vinka Mikulić, Kristina Ljubić, Vajdana Tomić

**Affiliations:** 1 Department of Laboratory Diagnostics, Clinical Hospital Centre Mostar, Mostar, Bosnia and Herzegovina; 2 School of Medicine, University of Mostar, Mostar, Bosnia and Herzegovina; 3 Department of Medical Biochemistry and Hematology, Faculty of Pharmacy and Biochemistry, University of Zagreb, Zagreb, Croatia; 4 Department of Obstetrics and Gynecology, Clinical Hospital Centre Mostar, Mostar, Bosnia and Herzegovina

**Keywords:** ferroportin, ferritin, iron, reference interval

## Abstract

**Introduction:**

Ferroportin (Fpn) is the only known iron exporter and plays an essential role in iron homeostasis. Serum concentrations of Fpn in health and/or diseased states are still mostly unknown. Therefore, the aim of this study was to determine the concentration of Fpn in the serum of women of reproductive age (WRA) for the first time, and to establish whether there is a difference in the concentration of Fpn according to ferritin status.

**Materials and methods:**

This research included 100 WRA (18-45 years, C-reactive protein (CRP) < 5 mg/L, hemoglobin > 120 g/L). Serum Fpn was measured using Enzyme Linked Immunosorbent Assay (ELISA) method on the analyzer EZ Read 800 Plus (Biochrom, Cambridge, UK). Reference interval was calculated using the robust method.

**Results:**

The median concentration of Fpn in the whole study group was 9.74 (5.84-15.69) µg/L. The subgroup with ferritin concentration > 15 µg/L had a median Fpn concentration 15.21 (10.34-21.93) µg/L, which significantly differed from Fpn concentration in the subgroup with ferritin concentration ≤ 15 µg/L (5.93 (4.84-8.36) µg/L, P < 0.001). The reference limits for the Fpn were 2.26-29.81 µg/L with 90% confidence intervals (CI) of 1.78 to 2.83 and 25.37 to 34.33, respectively.

**Conclusions:**

The proposed reference interval could help in the future research on iron homeostasis both in physiological conditions and in various disorders, because this is the first study that measured Fpn concentration in a certain gender and age group of a healthy population.

## Graphical abstract

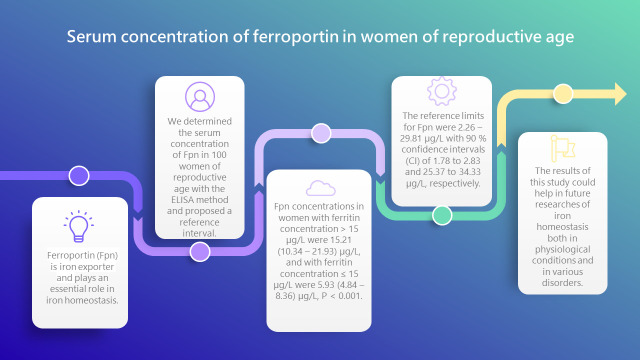

## Highlights

• Serum concentration of ferroportin in 100 women of reproductive age

• Serum ferroportin concentration in relation to ferritin concentration and recommended reference interval for ferroportin for women of reproductive age

• Ferroportin might become a potentially useful parameter in clinical states associated with disturbed iron homeostasis

## Introduction

Ferroportin (Fpn) participates in iron absorption from food, iron recycling and transport of stored iron. Ferroportin is expressed on enterocytes, hepatocytes and macrophages and it is the only known cellular iron exporter (*1*-*4*). In the structure of Fpn, there is a cytoplasmic loop that connects two bundles of transmembrane helices (N-loop and C-loop), and two bundles enclose a cavity through which iron exits. Ferroportin exports cellular iron by the mechanism of switching between an inward-open conformation that binds intracellular iron and an outward-open conformation that releases iron into the extracellular space (*5*). In addition, it functions as a hepcidin receptor, whose best-known physiological activity is binding to Fpn (*6*). Low hepcidin concentrations are associated with high concentrations of Fpn in iron-transporting tissues and rapid export of iron to the extracellular fluid and plasma. Otherwise, high concentrations of hepcidin in the circulation cause the removal of Fpn from the cell membranes, which inhibits the export of iron into the plasma (*7*). Iron is one of the most important essential elements for the human body, and most of it is consumed to produce erythrocytes. Iron deficiency, with or without anemia, can be absolute and functional and it affects many organ functions in the human body (*8*-*11*). Also, iron deficiency precedes anemia, and anemia in women of reproductive age (WRA) is a global public health problem and it is associated with regular blood loss (menstrual cycles), reduced iron stores, as well as increased needs, especially during pregnancy (*12*, *13*). The World Health Organization (WHO) has set hemoglobin (Hb) cut-off value for the classification of iron deficiency anemia in non-pregnant women, which is an Hb concentration below 120 g/L (*14*). The most used analyses for the iron status determination are serum iron (Fe) concentration, unsaturated iron binding capacity (UIBC), ferritin, and soluble transferrin receptors. Serum ferritin (SF) concentration is an indicator of the iron stores, when infection is not present (*15*). It is considered that the iron stores are depleted when the SF concentration is below 15 µg/L in adults (*16*). Determination of serum Fpn concentration might also be important as Fpn could be potentially used as indicator of iron status. Also, it could potentially help in the diagnosis, prognosis or treatment of anemias, but also disorders of iron overload such as ferroportin disease or hemochromatosis type 4 resulting from the *FPN1/SLC40A1* gene mutation (*17*). To date, the serum concentrations of Fpn are not known either in the healthy population, or in various disorders and diseases. Therefore, the aim of this study was to determine the concentration of Fpn in the serum of WRA for the first time and to establish whether there is a difference in the concentration of Fpn according to ferritin status. In addition, we proposed a reference interval for Fpn in the population of WRA in Bosnia and Herzegovina.

## Materials and methods

### Subjects

The present study was prospective and included 100 WRA (18-45 years). Those women were recruited at the Clinical Hospital Centre Mostar (CHCM), Bosnia and Herzegovina, from October 2022 to February 2023, as part of a regular determination of specialist tests that are not performed by primary health care. The CHCM provides health care for approximately 400,000 insured people, and the Department of Laboratory Diagnostics performs secondary and tertiary health care services, while primary health care is provided by about 10 laboratories in the territory of Herzegovina. The study was approved by the Ethics Committee of the CHCM (Ethical approving number 1036/21 at CHCM) in respecting to the Declaration of Helsinki. Written informed consent was obtained from all subjects after explaining the purpose of the study. The inclusion criteria after clinical examination were as follows: Hb > 120 g/L and C-reactive protein (CRP) < 5 mg/L. Exclusion criteria were anemia, inflammation, hypertension, diabetes, endocrine abnormalities, thyroid dysfunction, urinary infection, oncological diseases, autoimmune diseases, pregnancy, obesity, degenerative diseases and iron supplementation (*15*, *16*). We divided the women into two subgroups following the generally accepted WHO guidelines for the cut-off value of serum ferritin concentration: group with ferritin concentration ≤ 15 µg/L and group with ferritin concentration > 15 µg/L.

### Methods

In this study, the remains of serum and whole blood samples taken for routine laboratory tests at the Department of Laboratory Diagnostics of the CHCM were used. The blood samples were taken in the morning after overnight fasting. Venous blood samples were collected in a blood collection tube without anticoagulant (7.5 mL) and a K3-ethylenediaminetetraacetic acid (EDTA, 2.6 mL) tube (Sarstedt, Nümbrecht, Germany) with 21 G needles (Sarstedt, Nümbrecht, Germany). Serum samples for biochemical analyses were obtained by centrifuging whole blood without anticoagulants at 2320xg for 10 minutes, and the remaining part of serum after routine tests was stored at - 80 °C until determination of Fpn. C-reactive protein, Fe and UIBC concentrations (CRP Latex, Iron, UIBC; Beckman Coulter, Co. Clare, Ireland) were determined from serum on a Beckman Coulter DxC AU 700 chemistry analyzer (Beckman Coulter, Brea, USA) on the day of sample collection, as well as Hb concentration (Sulfolyser, Sysmex Europe GmbH, Norderstedt, Germany) which was determined from whole blood with EDTA on a Sysmex XN 1000 hematology counter (Wakinohama-Kaigandori Chuo-ku, Kobe, Japan). Total iron binding capacity (TIBC) was calculated, TIBC = Fe (µmol/L) + UIBC (µmol/L). Ferritin concentration (Vitros Ferritin Reagent Test, Ortho-Clinical Diagnostics, UK) was determined from a serum sample using the chemiluminescence method on a Vitros 3600 analyzer (Ortho-Clinical Diagnostics, Buckinghamshire, UK). The concentration of Fpn in serum was determined using the Enzyme Linked Immunosorbent Assay (ELISA) sandwich method (within 3 months from the day of storage at - 80 °C) on the EZ Read 800 Plus analyzer (Biochrom, Cambridge, UK). The Human Fpn Elisa Kit (AssayGenie, Cat.code HUES03226, Dublin 2, Ireland) was used to determine the concentration of Fpn. Standards or samples are added to the appropriate microplate wells and combined with the specific antibody. The absorbance is measured spectrophotometrically at 450 nm ± 2 nm. The concentration of human Fpn in samples can be calculated by comparing the absorbance of the samples to the standard curve. The calibration curve was obtained by serial dilution of the working solutions. The recommended dilution gradient is as follows: 20, 10, 5, 2.5, 1.25, 0.63, 0.31, and 0 µg/L (the last tube is regarded as a blank). This ELISA kit has no special quality control other than defined calibrator concentrations to obtain a calibration curve. The sensitivity of this test was 0.19 µg/L and inter and intra assay coefficients of variation (CV) for three samples (low, mid-range and high level) were 5.43, 5.19, 3.14% and 6.12, 4.50, 4.35%, respectively, provided by the manufacturer of the ELISA test. Detection range for this test was 0.31-20 µg/L. Each standard and sample was measured in duplicate. The recovery of Fpn in serum samples was in range 85-96%. Samples whose concentration was above 20 µg/L were diluted in a ratio of 1:2. The concentration of diluted samples was calculated from the standard curve, multiplied by the dilution factor. Assay linearities for 1:2 dilutions were in the range 88-100%.

### Statistical analysis

The results of the measurements are presented with appropriate measures of central tendency and dispersion, depending on the sample size and distribution. Shapiro-Wilk test was used to test the normality of data distribution. Data that followed a normal distribution are presented with the arithmetic mean and standard deviation (SD), and those that followed a non-normal distribution are presented with the median and interquartile range (IQR) (from first to the third quartile), except for age that is presented as the median and minimum-maximum. The Mann-Whitney U-test was used to compare two groups that did not follow a normal distribution. For the Fpn reference interval robust method was used as recommended by Clinical and Laboratory Standard Institute (CLSI) C28-A3c approved guideline when sample sizes are small, with less than 120 data. The robust method has the appeal of the parametric method because it does not require as many observations as the nonparametric procedure. With robust method, confidence intervals (CI) for reference limits are estimated using bootstrapping (percentile interval method) (*18*, *19*). In bootstrapping, sample data is used to create a large set of new “bootstrap” samples, simply by randomly taking data from the original sample (in this case 10,000 iterations). Data distribution histograms were visually inspected and the Tukey test was used for the identification of outliers. All differences with P < 0.05 are considered statistically significant. Data analysis was performed using MedCalc Statistical Software version 22.006 (MedCalc Software Ltd, Ostend, Belgium).

## Results

**Figure 1:**
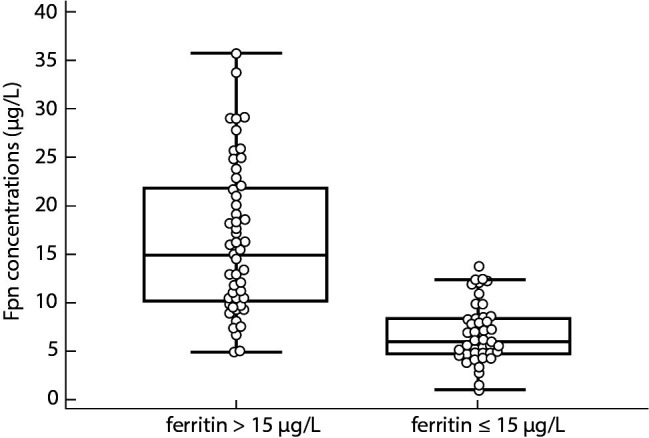
Serum ferroportin (Fpn) concentration in women of reproductive age with ferritin concentration > 15 µg/L and in women with ferritin concentration ≤ 15 µg/L.

**Figure 2:**
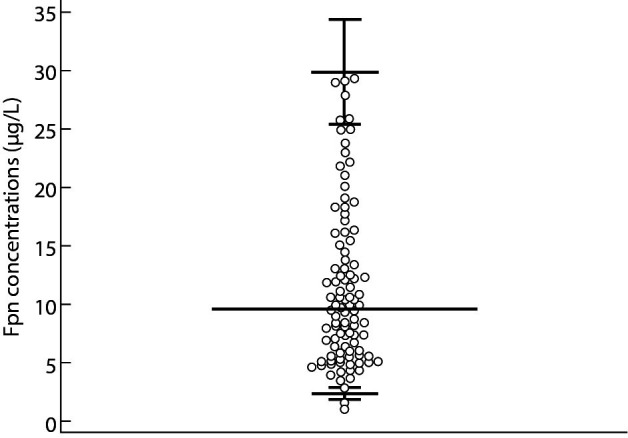
Graph of ferroportin (Fpn) concentration in women of reproductive age. Reference interval was determined by the robust method. The middle line represents the median.

The research included 100 WRA. The respective median age was 24 (18-44) years. Table 1 shows concentrations of iron status parameters (Fe, UIBC, TIBC and ferritin) as well as concentrations of Fpn, Hb and CRP in our study population. However, circulating concentration of Fpn differed statistically significantly when we subdivided our participants according to the concentration of ferritin that suggests if the iron stores are depleted or not. The median concentration of ferritin in the subgroup with concentration ≤ 15 µg/L was 11.1 (7.12-12.8) µg/L, and in the subgroup > 15 µg/L it was 21.2 (18.2-29.8) µg/L. Next, the median concentration of Fpn in the subgroup with ferritin concentration > 15 µg/L (N = 52) was 15.21 (10.34-21.93) µg/L and in the subgroup with ferritin concentration ≤ 15 µg/L (N = 48) it was 5.93 (4.84-8.36) µg/L (P < 0.001) (Figure 1). To define the reference interval with a robust method, we found that the lower limit with 90% CI was 2.26 (1.78 to 2.83), while the upper limit with 90% CI was 29.81 (25.37 to 34.33) (Figure 2).

**Table 1:** Concentrations of iron status parameters, Fpn, Hb and CRP

**Parameter**	**Result** **(N = 100)**
Fe (µmol/L)	15 (10-19)
UIBC (µmol/L)	49 ± 11
TIBC (µmol/L)	64 (58-70)
Ferritin (µg/L)	15.2 (11.1-21.5)
Fpn (µg/L)	9.74 (5.84-15.69)
Hb (g/L)	131 (126-136)
CRP (mg/L)	2.1 (0.9-2.8)
Results are expressed as mean ± standard deviation or median (interquartile range). Fe - iron. UIBC - unsaturated iron binding capacity. TIBC - total iron binding capacity. Fpn - ferroportin. Hb - hemoglobin. CRP - C-reactive protein.	

## Discussion

To the best of our knowledge, the results of this study are the first to present the concentration of ferroportin only in women of reproductive age. One study measured the concentration of Fpn in 60 women with breast cancer and 30 control women, between the ages of 30 and 70. Their results showed statistically significantly higher concentrations of Fpn (4.44 ± 1.20 ng/mL) in the control group compared to the tested group (2.47 ± 1.59 ng/mL) (*20*). This study does not list other possible diseases and disorders, as well as the analytical specifications of the test, so it is difficult to compare it with our study (*20*). Also, another study so far measured the serum concentration of Fpn in patients with schizophrenia and bipolar disorder and in healthy subjects (*21*). The control group consisted of only 42 people, both women (N = 23) and men (N = 19). They showed that the group of patients with schizophrenia had significantly higher concentrations of Fpn in serum (0.46 ± 0.22 ng/mL) than healthy subjects (0.26 ± 0.18 ng/mL) and patients with bipolar disorder (0. 30 ± 0.20 ng/mL) (*21*). In all three groups, serum concentrations of Fpn are lower compared to ours and their lower standard deviation is below the detection limit of our test.

We also demonstrated there is a statistically significant difference in the concentration of serum Fpn in women with ferritin concentration below 15 µg/L compared to those with ferritin concentration above 15 µg/L. Bosnia and Herzegovina does not have established data on the cut-off values of SF for its population. However, we would like to emphasize that the manufacturer of the reagents used in our research for SF specifically recommends reference values for women up to 45 years of age to be 7-133 ug/L, and for women over 45 years of age to be 14-295 ug/L. Therefore, according to the recommended reference values of the reagent manufacturer, all women who participated in this study have SF concentration within the reference interval. Also, the results of the study that compared two ferritin assays showed that Ortho-Clinical Diagnostics assay, that we used in this study, measures lower SF concentrations compared to assay of Abbott Architect, which shows that there is no standardization for immunochemical tests (*22*).

Previous researches measured parameters of iron status in women of reproductive age, mostly for the purpose of making a decision on iron supplementation, and they determined the cut-off value for SF in different populations and regions all around the world (*23*, *24*). The importance of interpreting iron status and iron homeostasis in pregnancy is particularly emphasized. More recent studies also include, in addition to the determination of routine iron parameters such as iron, Hb and ferritin, other parameters such as soluble transferrin receptors and hepcidin (*25*). Recently, in the focus of interest of many studies is the mechanism of action of the hepcidin-Fpn axis, which is the main regulator of iron homeostasis in the healthy population and in various diseases (*26*). In addition, different genetic errors can be the cause of iron homeostasis disorders, such as the iron overload or the development of anemia due to insufficient iron intake (*27*). Many studies have been devoted to the detection of Fpn gene/protein expression in different conditions such as adiposity, Alzheimer’s disease or breast cancer (*28*-*30*).

Our study is the first to include a larger number of women, and the first to determine the concentration of Fpn only in WRA. Also, for the first time, we proposed a reference interval in general for Fpn concentration. However, further research is needed on even larger number of subjects in order to determine the reference values in healthy population of both male and female as well as of different age groups. Today, only ELISA kits are available on the market for determining the concentration of Fpn from serum or plasma, and standardization of the method and verification of the test according to the CLSI EP15-A3 protocol for medical laboratory tests is required. Due to the role of iron in many functions in the body, it is important to know the concentration of Fpn in serum/plasma for better understanding of various disorders of iron homeostasis.

A limitation of this study is the possible latent iron deficiency in almost half of the women included in the study who have a ferritin concentration ≤ 15 µg/L.

## Data Availability

The data generated and analyzed in the presented study are available from the corresponding author on request.
